# Effect of smear layer pretreatment with EDTA and sodium hypochlorite on the dentin bond durability of universal adhesives

**DOI:** 10.3389/fdmed.2026.1781705

**Published:** 2026-03-18

**Authors:** Thanawat Ruaydee, Chantida Pawaputanon Na Mahasarakham, Vanthana Sattabanasuk, Pipop Saikaew

**Affiliations:** 1 Department of Operative Dentistry and Endodontics, Faculty of Dentistry, Mahidol University, Bangkok, Thailand; 2 Department of Restorative Dentistry, Faculty of Dentistry, Khon Kaen University, Khon Kaen, Thailand

**Keywords:** dentin bonding, EDTA, microtensile bond strength, smear layer, sodium hypochlorite, universal adhesive

## Abstract

**Aim:**

The purpose of this study was to investigate the effect of smear layer pretreatment on the dentin bond strength of two universal adhesives after 24 h and 6 months of water storage.

**Methods:**

In total, 92 extracted human third molars were used. Teeth were assigned to the following four groups: no treatment (SE), 37% phosphoric acid etching for 15 s (ER), agitation with 17% ethylenediaminetetraacetic acid (EDTA) for 30 s (ED), and agitation with 2.5% NaOCl for 60 s followed by Accel for 5 s (SA). Dentin surfaces were bonded with either Clearfil Universal Bond Quick (CUQ) or All-Bond Universal (ABU) and restored with resin composite (*n* = 10). Bonded specimens were sectioned into beams for microtensile bond strength (µTBS) testing and peripheral slabs for resin–dentin interface observation. Half of the beams were tested after 24 h, and the remaining beams after 6 months of water storage. An additional 12 teeth were used to observe smear layer surface changes. Scanning electron microscopy (SEM) was employed to evaluate smear layer morphology, resin–dentin interfaces, and failure modes. Data were analyzed using three-way ANOVA and Duncan’s multiple range test (*α* = 0.05).

**Results:**

Bond strength was significantly influenced by pretreatment method, storage time, and adhesive type (*p* < 0.05). ABU showed significantly higher µTBS than CUQ when applied using the ER technique. Applying CUQ using the ER technique and ABU using the SE technique resulted in a significant reduction in µTBS after 6 months. Smear layer pretreatment (ED and SA) demonstrated a bond strength similar to that achieved by applying the universal adhesives using the SE and ER techniques. A significantly higher µTBS in the ED and SA groups was only observed when compared with applying CUQ using the ER technique.

**Conclusion:**

Smear layer pretreatment with 17% EDTA or 2.5% NaOCl, followed by application of Accel™, provided a bond strength comparable to the conventional SE and ER techniques and provided a significantly higher bond strength than applying CUQ using the ER technique after both storage durations. These pretreatments are thus effective alternatives for dentin surface preparation before applying universal adhesives.

## 1 Introduction

Recently, universal adhesives have been brought to market as single-step systems. With the incorporation of versatile acidic functional monomers, universal adhesives can be bonded to a wide range of substrates, not only the tooth structures (1). Furthermore, due to their less aggressive pH, universal adhesives can be applied using different techniques, i.e., etch-and-rinse, self-etch, or selective enamel etch with phosphoric acid (2). As a consequence, their user-friendliness and simplification provide dentists with an alternative adhesive system for resin composite restoration.

Enamel is composed primarily of hydroxyapatite, and resin adhesion to enamel is considered more reliable when using the etch-and-rinse technique (3, 4). In contrast, dentin is an intrinsically hydrated tissue containing a dense network of fluid-filled tubules, making adhesion to dentin a much greater challenge. The presence of a smear layer has been reported to reduce dentin permeability by up to 86% (5). Phosphoric acid etching can completely remove the smear layer and aggressively demineralize the dentin (6), resulting in an increase of fluid flow onto the dentin surface (7). After rinsing, water should remain on the surface for the moist bonding technique used in the etch-and-rinse adhesive system. Pooled moisture (overly wet) or dry areas (overly dried) on the dentin surface can interfere with resin adhesion, e.g., incomplete infiltration of the resin at the bottom of the demineralized dentin zone (8, 9). In contrast, bonding to dentin using the self-etch technique is preferable and less technique-sensitive. By using self-etch primers, the smear layer is modified, and the underlying dentin is superficially demineralized (10). Adhesion is based on the simultaneous etching and priming of the dentin and incorporating the smear layer into the bonding layer. With this approach, the chance of unencapsulated collagen fibrils may be minimized (10). However, the smear layer may act as an obstacle that neutralizes the weak acidity of the primers and reduces the bonding effectiveness of the self-etch adhesives (11–13), especially with a bur-cut smear layer (14, 15).

The smear layer consists of both organic tissue debris and the inorganic material of tooth structures (16). To eliminate the potential effect of the smear layer on dentin adhesion, the layer can be removed by various chemical agents such as sodium hypochlorite and ethylenediaminetetraacetic acid (EDTA). Sodium hypochlorite is a routine irrigating solution for root canal treatment that mainly targets organic debris. Previous studies have shown the improved bonding performance of one-step self-etch adhesives using hypochlorous acid and sodium hypochlorite combined with a reducing agent on dentin (17–19). EDTA is a chelating agent that is used in the final flush before filling the root canal to remove the inorganic component in the root canal smear layer. Some studies also used EDTA prior to the application of simplified adhesives (20, 21). However, there is limited information about smear layer pretreatment before the application of recent universal adhesives. In our previous study, we investigated the immediate result of smear layer pretreatment with sodium hypochlorite followed by Accel and EDTA solution to improve the microtensile bond strength (µTBS) of universal adhesives (22). Therefore, it would be interesting to further evaluate whether smear layer pretreatment using various chemical agents influences the immediate and long-term adhesion of universal adhesives to dentin and also to investigate surface characteristics after smear layer pretreatments. The null hypothesis was that (1) no significant difference would be observed in dentin bond strength among the various smear layer pretreatment protocols; (2) no significant difference would be observed between immediate and long-term bond strength; and (3) no significant difference would be observed between different universal adhesives.

## 2 Materials and methods

### 2.1 Sample size calculation

The sample size calculation was conducted using the *F*-test family in G*Power (version 3.1.9.7; Heinrich Heine University Düsseldorf, Germany), with a statistical power of 80% and a significance level of 5%. The experimental design for evaluating microtensile bond strength comprised eight groups, based on two universal adhesives combined with four smear layer pretreatment protocols. Consequently, each group required 10 teeth, resulting in a total of 80 teeth for this component of the study, although the calculated minimum sample size was 73. This corresponded to the guideline for microtensile bond strength testing (23).

### 2.2 Tooth selection and bonding procedure

In total, 92 extracted human third molars collected from patients aged between 18 and 30 years old were used in this study. The teeth were free from caries, cracks, and restorations, and were used within 6 months after extraction. The teeth were kept in 0.1% thymol solution (M Dent, Bangkok, Thailand) until used. The protocol was ethically approved by the Faculty of Dentistry/Faculty of Pharmacy, Mahidol University Institutional Review Board, Thailand (IRB 2021/002.0302).

The universal adhesives used in this study were All-Bond Universal (ABU; Bisco, Schaumburg, IL, USA) and Clearfil Universal Bond Quick (CUQ; Kuraray Noritake Dental, Tokyo, Japan). The bonding protocols and chemical compositions of two adhesives are presented in Table 1.

**Table 1:** Chemical composition, pH, and application method of the universal adhesives used in this study.

Adhesive	Chemical composition	pH value	Application
All-Bond Universal (ABU; Bisco, Schaumburg, IL, USA)	10-MDP, phosphoric acid ester monomer, Bis-GMA, HEMA, ethanol, water, initiators	3.2	Apply two separate coats of adhesive with a rubbing motion for 15 s per coat, air-dry, and then light-cure using the curing unit for 10 s
Clearfil Universal Bond Quick (CUQ; Kuraray Noritake Dental, Tokyo, Japan)	Bis-GMA, HEMA, 10-MDP, hydrophilic amide monomer, ethanol, water, camphorquinone, colloidal silica, silane coupling agent, sodium fluoride	2.3	Apply the adhesive with rubbing motion for 5 s, air-dry, and then light-cure using the curing unit for 10 s

### 2.3 Evaluation of the effect of smear layer pretreatment on the µTBS of universal adhesives

In total, 80 human third molars were used in this part. They were randomly allocated to eight experimental groups (*n* = 10) using simple randomization with equal allocation. Their roots were removed at 1 mm below the cemento-enamel junction to expose the pulp chambers using a slow-speed water-cooled diamond saw (Isomet, Buehler Ltd., Lake Bluff, IL, USA). Occlusal enamel was removed with a model trimmer and the teeth were ground with 600-grit silicon carbide (SiC) paper (Carbimet, Buehler Ltd.) under running water to obtain flat coronal dentin surfaces. The exposed occlusal dentin surfaces were further ground using a medium-grit diamond bur (Jota, Rüthi, Switzerland; mean abrasive particle size 90–106 µm) at high speed (320,000–350,000 rpm) with five light pressure strokes (14). A new bur was used after five surfaces to create clinically relevant, uniform surfaces. A single operator was trained to standardize the applied manual pressure using an analytical balance, corresponding to approximately 100 g of force during manipulation (24). Then, dentin surfaces were randomly divided into four groups according to different smear layer modification methods as follows:

- Group SE: No surface treatment—the universal adhesives were used in the self-etch strategy as per the manufacturer’s instructions.
- Group ER: Phosphoric acid etching was applied for 15 s, followed by rinsing with distilled water for 30 s. The universal adhesives were used as per the manufacturer’s instructions in the etch-and-rinse strategy.
- Group ED: 17% EDTA solution (approximately 0.05 mL) was dropped onto the dentin surface, and then rubbed with a microbrush for 30 s. The dentin surfaces were rinsed with distilled water for 30 s and air-dried. One of the universal adhesives was applied afterward.
- Group SA: 2.5% NaOCl solution (approximately 0.05 mL) was dropped onto the dentin surface, and then rubbed with a microbrush for 60 s. The surfaces were rinsed with distilled water for 30 s and air-dried. One drop of Accel (approximately 0.05 mL) was applied for 5 s, followed by air drying. One of the universal adhesives was applied afterward.

After smear layer pretreatment, each group was further divided into two subgroups of 10 teeth according to the universal adhesive used (All-Bond Universal or Clearfil Universal Bond Quick). Resin-based composite Clearfil AP-X shade A3 (Kuraray Noritake Dental) was placed on the treated dentin surface in 2-mm incremental layers. Light polymerization was conducted on each increment separately to a total of 4 mm in height using a light-curing unit (Bluephase G2, Ivoclar Vivadent, Schaan, Lichtenstein) with light intensity not less than 1,000 mW/cm^2^. After this, all the bonded teeth were stored in distilled water at 37°C for 24 h.

### 2.4 Evaluation of µTBS

Each tooth was cut on the x and y axes using a slow-speed diamond saw under water cooling. Using the non-trimming technique, six to eight resin-dentin beams, approximately 1 mm × 1 mm × 6 mm, from the central part of each tooth were derived and used in the µTBS test (25). Half of the resin-dentin beams were randomly selected and subjected to the 24-h bond strength test. The remaining resin-dentin beams were stored in distilled water for a further 6 months with regular media replacement biweekly and were used in the 6-month µTBS evaluation (23).

Specimens were fixed on an experimental jig in the µTBS test using cyanoacrylate glue (Model Repair II Blue; Dentsply-Sankin, Tochigi, Japan). The µTBS test was performed using a universal testing machine (Lloyd Testing Machine, Model LR 10K, Lloyd Instruments, Fareham Hanth, UK) with a cross-head speed of 1 mm/min. Data were recorded and expressed in MPa. The collected data were converted to the arithmetic mean of an individual tooth for further statistical analysis. Pre-testing failure was included in the calculation using a given value of 0 MPa (23).

### 2.5 Mode of failure evaluation

The fractured surfaces of both the resin and dentin sides were left to dry overnight. Then, the surfaces were coated with gold (Sputter.SPI Structure Prope, West Chester, PA, USA) and examined using scanning electron microscopy (SEM; JSM-5410 LV, JEOL, Tokyo, Japan) at 80× magnification to determine the mode of failure. The mode of failure was classified into one of the following four types: adhesive failure, cohesive failure in the resin, cohesive failure in the dentin, and mixed failure (23, 26).

### 2.6 Evaluation of the effect of smear layer modification by SEM

In total, 12 teeth were cut parallel to the occlusal surface at the mid-coronal part into 2-mm thick disk-shaped specimens using a slow-speed water-cooled diamond saw (Isomet™, Buehler Ltd.) The dentin disc surfaces were ground using a medium-grit diamond bur (Jota) in the same manner as the µTBS test. The dentin disk specimens were subjected to different smear layer modification methods, with two specimens per group.

- Group A: No treatment.
- Group B: Application of Clearfil Universal Bond Quick using the self-etch technique.
- Group C: Application of All-Bond Universal using the self-etch technique.
- Group D: Application of 17% EDTA solution by rubbing for 30 s and then rinsing with distilled water for 30 s.
- Group E: Application of 2.5% NaOCl solution by rubbing for 60 s, rinsing with distilled water for 30 s, and air-drying. Accel was applied for 5 s, followed by air drying.
- Group F: Application of 37% phosphoric acid for 15 s, then rinsing with distilled water for 30 s.

In groups A and B, after the application of the universal adhesives, the dentin disks were rinsed with 100% acetone for 1 min to remove the adhesive (27). Then, the disk specimens were fixed in 2.5% glutaraldehyde and 0.1 M Sorensen phosphate buffer for 24 h. The specimens were rinsed with the same buffer. The disks were then dehydrated using increasing concentrations of ethanol. After the dehydration process, the dentin disks were immersed in hexamethyldisilane (HMDS) for 10 min and left overnight (28). After drying, the disks were gold sputter-coated and observed using SEM.

In groups C–F, the specimens were fixed in 2.5% glutaraldehyde and 0.1 M Sorensen phosphate buffer for 24 h. Then, the disks were rinsed with the same buffer. After this, the disks were dehydrated and immersed in HMDS as described previously.

### 2.7 Observation of the resin–dentin interface

One resin–dentin slab from the peripheral part of the tooth that was prepared for the µTBS test was used for the resin–dentin interface observation (29). Three slabs were randomly selected and examined after 24 h, while the remaining specimens were stored in distilled water for 6 months. After storage, three additional slabs were randomly selected and examined in the long-term evaluation.

The specimens were placed inside an acrylic ring that was attached to double-sided tape and embedded in epoxy resin. The epoxy-embedded specimens were ground using #600 SiC paper for 60 s under running water, followed by #800, #1,000, #1,200, #1,500, #2,000, and #3,000 grit paper in the same manner with ultrasonic cleansing between steps. A glass plate was used as a grinding base to help maintain sample flatness. Subsequently, the specimens were immersed in 1 M hydrochloric acid for 30 s and 5% sodium hypochlorite for 5 min, followed by water rinsing and air drying. Then, all the specimens were coated with gold and observed under SEM (JSM 6610LV, JEOL Inc., Peabody, MA, USA) (30).

### 2.8 Statistical analysis

The bond strength data were calculated and the distribution of the data was analyzed using the Shapiro–Wilk test. Homogeneity of variance was analyzed using Levene’s test. As the data were normally distributed, three-way ANOVA and Duncan’s multiple range test were used to analyze and detect any significant differences between the experimental groups. All the analyses were performed at a 95% confidence level.

## 3 Results

### 3.1 Evaluation of the effect of smear layer pretreatment using scanning electron microscopy

**Figure 1:**
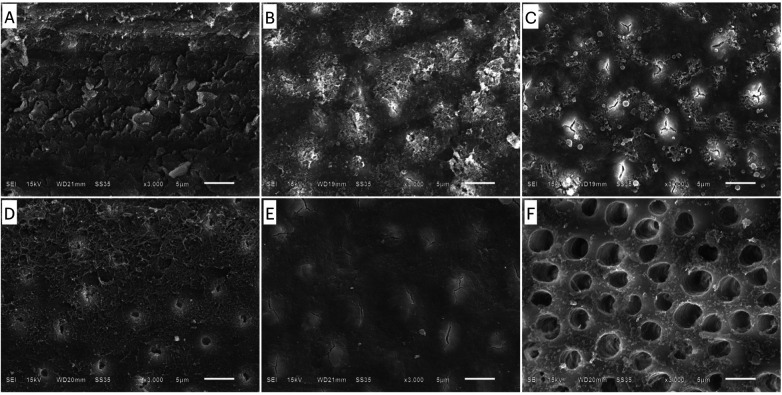
SEM images showing the surface characteristics of dentin with different pretreatments: **(A)** bur-cut smear layer; **(B)** CUQ agitated using the self-etch technique; **(C)** ABU agitated using the self-etch technique; **(D)** agitation with EDTA for 30 s; **(E)** agitation with NaOCl for 60 s; and **(F)** etching with phosphoric acid for 15 s.

Figure 1 illustrates the surface characteristics of the smear layer with different pretreatments. Figure 1A illustrates a bur-cut smear layer covering the dentin surface. When applying the universal adhesives using the self-etch technique, the superficial smear layer was demineralized, as illustrated in Figures 1B,C. Smear layer pretreatment with 2.5% NaOCl agitation for 60 s or 17% EDTA agitation for 30 s partially removed the covering smear layer, but with different characteristics. In the underlying dentin structure, the opening of dentinal tubules and collagen frameworks were found when using 17% EDTA agitation (Figure 1D). Dentin surface modification was less aggressive when using NaOCl. The smear layer was removed, but the dentinal tubules were still occluded (Figure 1E). Figure 1F shows the complete removal of the smear layer and aggressive demineralization due to phosphoric acid etching. The dentinal tubules were wide open and collagen frameworks were observed deep in the tubules.

### 3.2 Evaluation of the effect of smear layer pretreatment on the microtensile bond strength of universal adhesives

**Figure 2:**
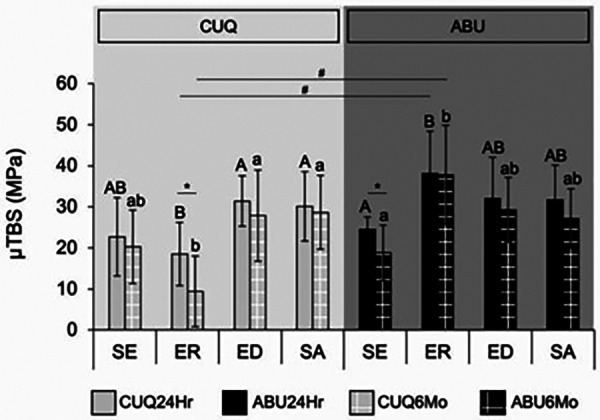
μTBS of dentin bonded with CUQ and ABU adhesives after 24 h and 6 months of water storage under different pretreatment conditions: self-etch technique (SE), etch-and-rinse technique (ER), EDTA pretreatment (ED), and sodium hypochlorite pretreatment (SA). Bars represent mean ± standard deviation. Different uppercase letters indicate significant differences among the pretreatment groups after 24 h, while lowercase letters indicate significant differences after 6 months of storage. The symbol * denotes significant differences between storage periods, and # indicates significant differences between adhesives (*p* < 0.05).

**Figure 3:**
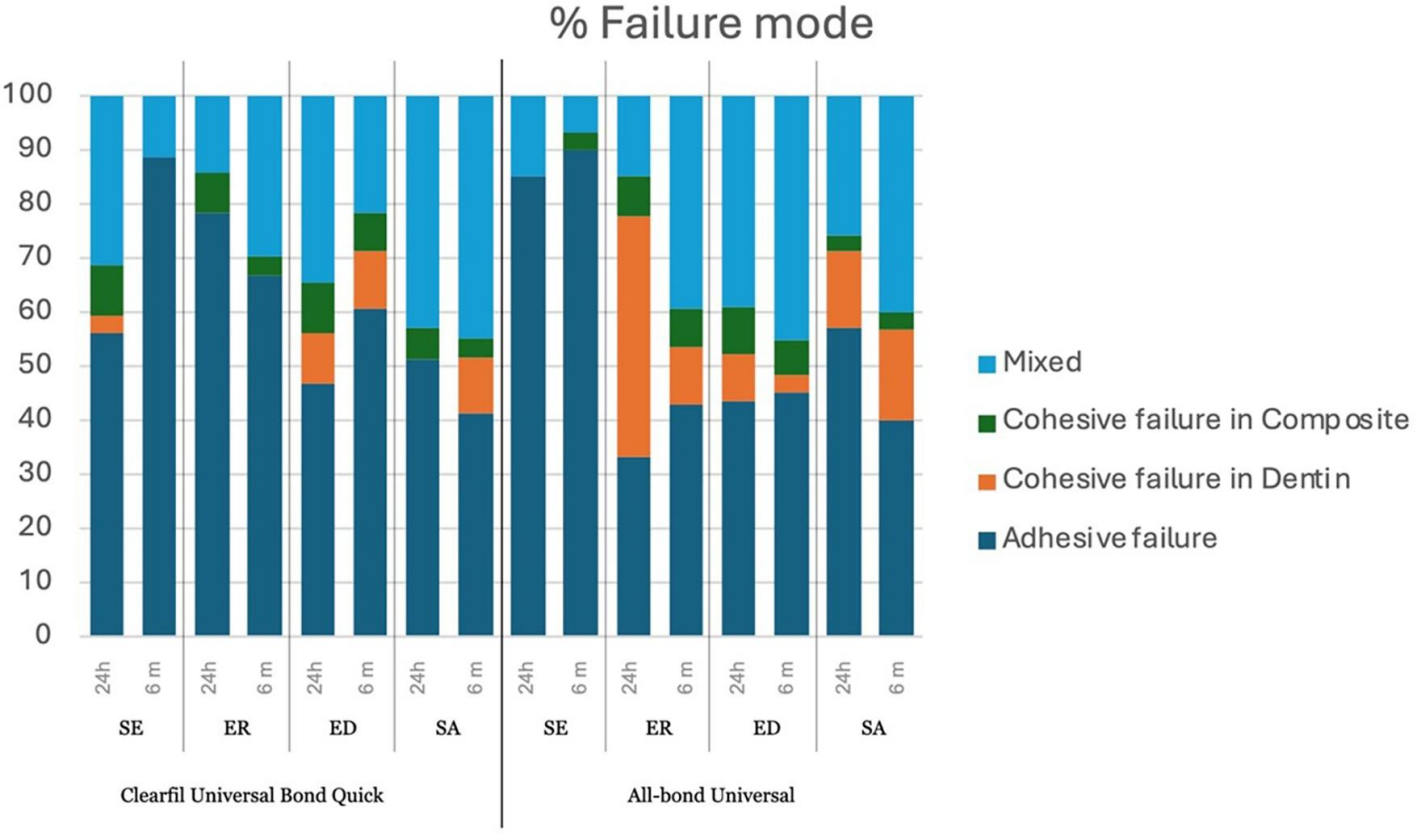
Percentage of failure mode in all the experimental groups. Self-etch technique, SE; etch-and-rinse technique, ER; EDTA pretreatment, ED; sodium hypochlorite pretreatment, SA.

The microtensile bond strength values and standard deviations in all the experimental groups are shown in Figure 2. The three-way ANOVA revealed no significant interaction between storage time and the other factors (*p* > 0.05). The two-way ANOVA revealed the significant effect of adhesive, treatment method, and their interactions after both storage durations (*p* < 0.05). The µTBS values of CUQ and ABU after both storage durations demonstrated similar trends. For CUQ, the ED and SA groups exhibited significantly higher µTBS compared with the ER group. In contrast, the highest bond strength was observed in ABU when using ER, which was significantly higher than that in the SE group. A higher percentage of cohesive failures, especially in dentin, was also found (Figure 3). Smear layer modifications (ED and SA) did not produce a significant effect in ABU, with either SE or ER (*p* > 0.05). When comparing the adhesives, ABU demonstrated significantly higher µTBS than CUQ when bonded using the ER technique. A paired *t*-test further revealed the significant effect of storage time, with a significant decrease in bond strength after 6 months of water storage for CUQ with ER and for ABU with SE (*p* < 0.05).

### 3.3 Observation of the resin–dentin interface

**Figure 4:**
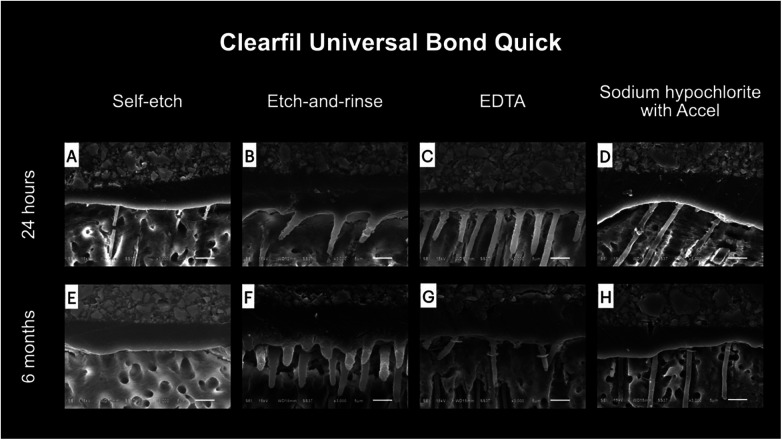
Representative SEM images of the resin–dentin interface of Clearfil Universal Bond Quick under different smear layer pretreatments at 24 hours **(A–D)** and 6 months **(E–H)**. Bonding in self-etch mode demonstrated limited resin penetration and short resin tags **(A, E)**. Etch-and-rinse mode showed more pronounced resin penetration with funnel-shaped resin tags **(B, F)**. Pretreatment with 17% EDTA produced distinct cylindrical resin tags extending into dentinal tubules **(C, G)**. Pretreatment with 2.5% NaOCl followed by Accel™ also resulted in cylindrical resin tags with moderate penetration **(D, H)**. No notable morphological differences were observed between 24 hours and 6 months.

**Figure 5:**
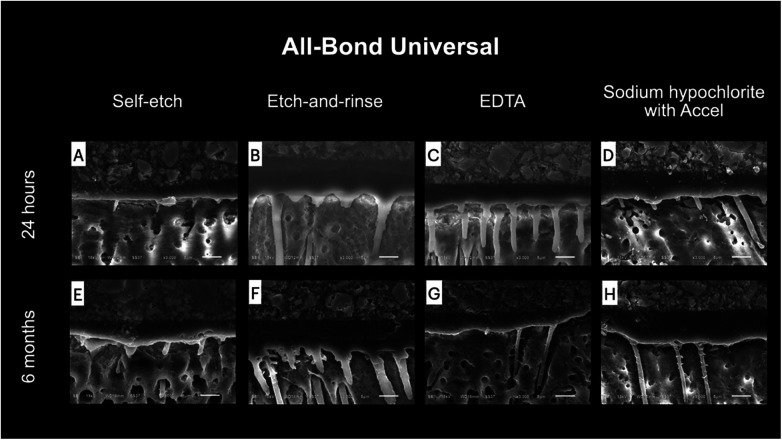
Representative SEM images of the resin–dentin interface of All-Bond Universal under different smear layer pretreatments at 24 hours **(A–D)** and 6 months **(E–H)**. Application in self-etch mode produced a thin hybrid layer with limited resin penetration and short resin tags **(A, E)**. Etch-and-rinse mode resulted in deeper resin infiltration and prominent funnel-shaped resin tags **(B, F)**. Pretreatment with 17% EDTA generated cylindrical resin tags extending into dentinal tubules **(C, G)**. Pretreatment with 2.5% NaOCl followed by Accel™ also produced cylindrical resin tags with moderate penetration **(D, H)**. Comparable interfacial morphology was observed between 24 hours and 6 months.

Representative SEM images of the resin–dentin interface after using different smear layer pretreatment methods are shown in Figures 4, 5. No notable difference between 24 h and 6 months was detected. Limited resin penetration was observed in both ABU and CUQ when applied using the self-etch technique. More pronounced resin tags were observed when the universal adhesives were applied using the etch-and-rinse technique or after applying smear layer pretreatments. The resin tags found in the etch-and-rinse group were funnel-shaped, whereas the resin tags in the 17% EDTA and 2.5% NaOCl groups were cylindrically shaped.

## 4 Discussion

According to the findings of this study, smear layer pretreatment and applying a universal adhesive using the ER technique significantly influenced dentin bond strength. Therefore, the first null hypothesis was rejected. After 6 months of water storage, a significant reduction in µTBS was observed in CUQ when applied using the ER technique, leading to the rejection of the second null hypothesis. Moreover, the µTBS values of ABU were significantly higher than those of CUQ in the ER group, irrespective of storage time. Consequently, the third null hypothesis was also rejected.

In this study, the effect of smear layer pretreatment on the dentin bond strength was investigated using two universal adhesives, CUQ and ABU, as representatives of mild and ultra-mild pH adhesives, respectively (31). The aim of smear layer pretreatment is to facilitate resin penetration into dentin by partially removing the smear layer. The composition of the smear layer includes organic components and inorganic components. Therefore, in this study, the smear layer was either agitated with 2.5% NaOCl solution or 17% EDTA solution to interact with the organic and inorganic components separately. Some studies have investigated rinsing with these chemical agents to modify the smear layer (17–19, 21, 32). However, in this study, these chemical agents were agitated with a microbrush to enhance smear layer removal and control the solution within the designated area.

The bond strengths of CUQ when applied using the SE and ER techniques were not significantly different. This result corresponded with other studies that demonstrated similar bond strengths when universal adhesives were applied using different etching techniques (2, 33–35). The smear layer pretreatments in the SA and ED groups significantly improved the bond strength of CUQ when compared to the ER group. This result is supported by our SEM images. Partial smear layer removal by these agents was observed. In addition, prominent resin tags after adhesive application in both the smear layer pretreatment groups were observed. Thus, smear layer pretreatments are beneficial for CUQ in terms of bond strength improvement and better bonding interface quality.

ABU is unique due to its higher pH than other universal adhesives (1). It has been reported that the performance of this adhesive is compromised when applied using the SE technique due to its weak acidity (36–39). Moreover, the smear layer removal and resin penetration of this mild self-etch adhesive were limited, especially when encountering a dense and compact bur-cut smear layer (13, 29, 40). In contrast, the application of ABU using the ER technique demonstrated the highest bond strength, which corresponded with the highest percentage of cohesive failure. Applying ABU using the ER technique also demonstrated significantly higher bond strength when compared to using the SE technique. This result is in line with previous studies (36–38). Smear layer pretreatments with NaOCl and EDTA demonstrated no significant difference in µTBS for ABU applied using the SE technique. Despite the lower acidity with a pH of 3.2, ABU positively influences bond quality due to its double-layer application. Replenishing the universal adhesive enhances the bonding performance compared with the single-layer application method (41). Moreover, ABU contains no filler and thus facilitates resin penetration through the smear layer (42, 43), which may explain why it provided comparable bond strength in the SE and smear layer pretreatment groups.

Studies have investigated the effect of smear layer pretreatments on the immediate dentin bond strength of simplified adhesives (18, 32, 44, 45). However, no investigation had been conducted to evaluate the effect of smear layer pretreatment on long-term bond durability. Therefore, in this study, the specimens were kept in water storage for 6 months and a decrease in µTBS in all groups was observed. However, a statistically significant reduction in bond strength was only detected in the CUQ and ER group. The lowest bond strength of CUQ when applied using this technique corresponded with the highest incidence of pre-test failure. This could be the consequence of ineffective resin penetration into deeply etched dentin. Thus, degradation of the resin–dentin bond could be expected as this phenomenon can usually be found in etch-and-rinse adhesives (8). In contrast, applying ABU using the ER technique resulted in the highest bond strength after both storage durations, likely due to the previously described double application of the adhesive (15, 41). The monomer properly penetrates the etched dentin and forms complete hybridization. The resin-encapsulated collagen fibrils are protected from the degradation process (8, 9, 46). Where the complete infiltration of adhesive into the exposed collagen fibril network occurs, no aqueous solution within the hybrid layer is detected. Hydrolysis of the resin matrix and enzymatic degradation, such as matrix metalloproteinases (MMPs), is minimized and the stability of the resin–dentin interface is then expected in the long term.

The smear layer pretreated groups had improved long-term bond strength for both adhesives. Significantly higher µTBS were observed in CUQ after the ED and SA pretreatments compared with the ER group, whereas for ABU, significantly higher values were observed in the ED pretreatment group compared with the SE group. The effect of the smear layer pretreatments can be explained separately. EDTA is a chelating agent that removes inorganic minerals from the smear layer and partially demineralizes the dentin. Furthermore, EDTA has the potential to inhibit MMPs. In contrast, NaOCl has proteolytic activity that dissolves organic debris. Combined with the Accel application, NaOCl-treated dentin has a less hybridized smear layer and minimized nanoleakage (17). Smear layer removal with EDTA was more effective than NaOCl, despite its shorter application time. The collagen fibril and dentinal tubules orifices were clearly observed.

When comparing the two universal adhesives, similar bond strengths were observed when they were applied using the SE technique. This can be explained by the pH of the adhesives and the application technique. Due to the higher pH of ABU, the demineralization effect may be inadequate, with limited resin penetration. The bond strength of ABU is expected to be lower than that of CUQ. However, the double application, as indicated by the manufacturer's instructions, may have improved the etching effect of ABU (15, 41).

Unlike the SE technique, applying ABU using the ER technique resulted in significantly higher bond strength than when applying CUQ. This could be explained by the difference in application technique. After phosphoric acid etching, complete smear layer removal with deep demineralization of underlying dentin occurs. Therefore, the etch-and-rinse approach is technique-sensitive in achieving complete hybridization with the adhesive. CUQ is indicated to be applied for up to 5 s, while the instruction for ABU indicates a double application of the adhesive for 15 s each. This technique may ensure monomer infiltration into deep demineralized dentin. A significantly higher bond strength for CUQ with a prolonged application time of up to 20 s has been previously reported (35). Therefore, it may be interesting to evaluate applying CUQ using the ER technique with a prolonged application time.

Another possible explanation is the viscosity of the uncured adhesive. Regarding the incorporation of filler into both the tested adhesives, CUQ contains colloidal silica filler, while ABU has no filler. Although the addition of filler may improve the mechanical properties of the adhesive layer, its viscosity may increase due to filler agglomeration (47). This could also prevent the proper infiltration of the adhesive into the deeply etched dentin, resulting in the deficient hybrid layer and inferior bonding performance of CUQ compared with ABU found in the current investigation.

As an *in vitro* investigation, the study did not fully replicate intraoral conditions, such as thermal cycling, masticatory stress, pH fluctuations, saliva contamination, and enzymatic activity, which may influence long-term bond performance. Regarding long-term bond strength, there was a bond strength reduction trend in almost all the groups. A longer storage period may be required to discriminate bond durability between the groups. To elucidate the long-term effect at the resin–dentin interface, silver staining that reveals nanoleakage in the adhesive and the hybrid layer would be illustrative.

## 5 Conclusion

Smear layer pretreatment enhanced the dentin bond strength and durability of Clearfil Universal Bond Quick but had no significant effect on All-Bond Universal, for which the etch-and-rinse technique remained superior.

## Data Availability

The raw data supporting the conclusions of this article will be made available by the authors, without undue reservation.
